# Lifestyle‐related risk factors and trajectories of work disability over 5 years in employees with diabetes: findings from two prospective cohort studies

**DOI:** 10.1111/dme.12787

**Published:** 2015-05-15

**Authors:** M. Virtanen, M. Kivimäki, M. Zins, R. Dray‐Spira, T. Oksanen, J. E. Ferrie, A. Okuloff, J. Pentti, J. Head, M. Goldberg, J. Vahtera

**Affiliations:** ^1^ Finnish Institute of Occupational HealthHelsinkiTurku and TampereFinland; ^2^ Department of Epidemiology and Public HealthUniversity College LondonLondonUK; ^3^ Population‐Based Cohorts UnitInserm UMS 011VillejuifFrance; ^4^ University VersaillesSaint Quentin en YvelinesFrance; ^5^ INSERM, UMR_S 1136Pierre Louis Institute of Epidemiology and Public HealthDepartment of Social EpidemiologyParisFrance; ^6^ Sorbonne UniversitésUPMC Univ Paris 06 UMR_S 1136Pierre Louis Institute of Epidemiology and Public HealthDepartment of Social EpidemiologyParisFrance; ^7^ School of Community and Social MedicineUniversity of BristolBristolUK; ^8^ Department of Public HealthUniversity of Turku and Turku University HospitalTurkuFinland

## Abstract

**Aims:**

To examine work disability trajectories among employees with and without diabetes and identify lifestyle‐related factors associated with these trajectories.

**Methods:**

We assessed work disability using records of sickness absence and disability pension among participants with diabetes and age‐ sex‐, socio‐economic status‐ and marital status‐matched controls in the Finnish Public Sector Study (1102 cases; 2204 controls) and the French GAZEL study (500 cases; 1000 controls), followed up for 5 years. Obesity, physical activity, smoking and alcohol consumption were assessed at baseline and the data analysed using group‐based trajectory modelling.

**Results:**

Five trajectories described work disability: ‘no/very low disability’ (41.1% among cases and 48.0% among controls); ‘low–steady’ (35.4 and 34.7%, respectively); ‘high–steady’ (13.6 and 12.1%, respectively); and two ‘high–increasing’ trajectories (10.0 and 5.2%, respectively). Diabetes was associated with a ‘high–increasing’ trajectory only (odds ratio 1.90, 95% CI 1.47–2.46). Obesity and low physical activity were similarly associated with high work disability in people with and without diabetes. Smoking was associated with ‘high–increasing’ trajectory in employees with diabetes (odds ratio 1.88, 95% CI 1.21–2.93) but not in those without diabetes (odds ratio 1.32, 95% CI 0.87–2.00). Diabetes was associated with having multiple ( ≥ 2) risk factors (21.1 vs. 11.4%) but the association between multiple risk factors and the ‘high–increasing’ trajectory was similar in both groups.

**Conclusions:**

The majority of employees with diabetes have low disability rates, although 10% are on a high and increasing disability trajectory. Lifestyle‐related risk factors have similar associations with disability among employees with and without diabetes, except smoking which was only associated with poorer prognosis in diabetes.

## What's new?

- We examined trajectories of work disability among people with and without diabetes.
- Five trajectories describing disability level at the beginning of follow‐up and its development over 5 years were identified: ‘no/very low disability’, ‘low–steady’, ‘high–steady’ and two ‘high–increasing’ trajectories.
- The majority of employees with and without diabetes had low‐disability trajectories.
- Diabetes was associated with ‘high–increasing’ disability trajectories, although this affected only 10% of the population with diabetes.
- Obesity and physical inactivity, irrespective of diabetes, and smoking among employees with diabetes were associated with adverse disability trajectories.

**What's new?**

- We examined trajectories of work disability among people with and without diabetes.
- Five trajectories describing disability level at the beginning of follow‐up and its development over 5 years were identified: ‘no/very low disability’, ‘low–steady’, ‘high–steady’ and two ‘high–increasing’ trajectories.
- The majority of employees with and without diabetes had low‐disability trajectories.
- Diabetes was associated with ‘high–increasing’ disability trajectories, although this affected only 10% of the population with diabetes.
- Obesity and physical inactivity, irrespective of diabetes, and smoking among employees with diabetes were associated with adverse disability trajectories.

## Introduction

Diabetes is a common chronic condition among working‐age populations and is associated with an increased risk of macro‐ and microvascular complications 1, reduced functional capacity, including depression and fatigue 2, 3, sickness absence 4, 5, 6, 7, early retirement and disability pension 8, 9. With the increasing burden of diabetes worldwide 10, identification of factors that influence working capacity among people with diabetes is increasingly important.

Obesity, physical inactivity, smoking and high alcohol consumption have generally been shown to be associated with sickness absence and work disability pensions in working populations 11, 12, 13, 14, 15, 16. A healthy lifestyle has also been shown to be very important for the management of diabetes and prevention of diabetes‐related adverse complications 1; however, it is not known which types of trajectories can be identified and which lifestyle‐related risk factors contribute most to trajectories of work disability among employees with diabetes. It is also not known whether disability trajectories are similar in different occupational cohorts and among employees with and without diabetes. In the present study we address these outstanding questions using survey and register data from two occupational cohort studies.

## Participants and methods

### Research design and setting

This study included two ongoing prospective study cohorts: The Finnish Public Sector study (FPSS) 5, 13, coordinated by the Finnish Institute of Occupational Health, is a prospective cohort of employees working in 10 towns and 21 hospitals. The Ethics Committee of the Hospital District of Helsinki and Uusimaa approved the study. The baseline for the present study was in 2004 (Fig. S1) when a total of 48 076 participants responded to a survey (response rate 66%). This baseline was chosen because complete sickness absence data for follow‐up were available from 2005. In addition to the survey, health records between 2001 and 2004 (see description below) were used to identify diabetes cases, yielding a total of 1359 diabetes cases. Follow‐up of sickness absence was from 1 January 2005 to 31 December 2009. During the follow‐up, 14 employees died and 243 retired, resulting in an analytical sample of 1102 diabetes cases. Using the same procedure as for diabetes cases, we randomly selected two controls without diabetes and with 5‐years follow‐up of work disability (n = 2204) matched by age, sex, socio‐economic status and marital status. Covariates were derived from the survey and registers in 2004.

The GAZEL cohort study, established in 1989, comprises employees from the French national gas and electricity company Electricité de France‐Gaz de France 4, 8. The study was approved by the Inserm Ethics committee and all participants gave informed consent. At baseline, 20 625 employees (73% men), aged 35–50 years, participated (response rate 45%), and follow‐up relied mainly on an annual survey including a questionnaire. Of the participants who responded to at least one survey between the years 1989 and 2003 (Fig. S1), 914 were identified as having diabetes. Of these, 15 died, 363 retired and 36 left the organization before the end of the 5‐year follow‐up, resulting in an analytical sample of 500. Diabetes was already present at study outset in 1989 (Survey 1) for some of the participants. For these, follow‐up started immediately after Survey 1. In other participants, diabetes was detected after the study had started (e.g. at Survey 2). For these participants, follow‐up started immediately after diabetes was detected. Covariates were collected from the most recent survey. Two control subjects without diabetes and with 5 years’ follow‐up of work disability (*n *= 1000) for each diabetes case were randomly derived from the baseline (1989) survey, matched by age, sex, socio‐economic status and marital status. All participants were followed‐up for 5 calendar years.

### Measures

In the FPSS, identification of diabetes cases was based on national registers of purchased diabetes medicines (oral medication or insulin) and entitlements to special reimbursements for their costs by the Social Insurance Institution of Finland which covers all permanent residents. To be eligible for this register, a patient's condition must meet explicit predefined criteria (diabetes which has not been responsive to lifestyle intervention and needs long‐term antidiabetic treatment). Participants with diabetes were also identified from responses to a survey question on doctor‐diagnosed diabetes. Data from all these sources were compiled to identify employees with diabetes. In the GAZEL study, participants with diabetes were identified from responses to a checklist of > 50 chronic conditions in annual surveys.

In both cohorts, BMI was calculated from self‐reported height and weight to identify obese (BMI ≥ 30 kg/m^2^) participants. Low physical activity was defined as < 0.5 h of vigorous physical activity (e.g. brisk walking, jogging and running) per week (FPSS) and as no sports activities (GAZEL). Smoking status was categorized as current smoker vs. non‐smoker. The average amount of beer, wine and spirits consumed per week (FPSS) or day (GAZEL) was transformed into units of alcohol per week. Risky alcohol use was defined as ≥ 22 units/week (men) or ≥ 15 units/week (women) 17.

Work disability was based on annual number of days on sickness absence and work disability pension over 5 years. In Finland (FPSS cohort), the national sickness allowance scheme covers sickness absence of > 9 days. Work disability pension can be granted after ~ 1 year of sickness absence. For the FPSS cohort, we obtained data on sickness absence and work disability pension between 1 January 2005 and 31 December 2009. In the GAZEL cohort, employees were covered by a company‐run insurance scheme. The policy regarding long‐term sickness absence was to grant a disability pension after 5 years of absence. We obtained sickness absence and work disability pension records (1 January 1990 to 31 December 2008) from Electricité de France‐Gaz de France. All these records included the first and last dates (if relevant) of all absences and disability pensions. For each employee, we computed the annual sum of disability days for the 5‐year follow‐up period.

Socio‐demographic baseline covariates were age, sex, socio‐economic status (occupational grade) and marital status (married or cohabiting vs single, divorced or widowed). Comorbid physical diseases were obtained at baseline. In the FPSS cohort, data on comorbid disease (chronic hypertension, ischaemic heart disease, heart failure, rheumatoid arthritis, asthma or chronic obstructive pulmonary disease) were based on entitlements to special reimbursement for medication. In the GAZEL cohort, information on the corresponding diseases (hypertension, myocardial infarction, angina, stroke, osteoarthritis, rheumatoid arthritis, asthma) was based on survey responses.

### Statistical analysis

We used group‐based trajectory modelling, implemented in sas version 9.4, to identify clusters of individuals (trajectory groups) who have followed a similar developmental trajectory for work disability in their annual count of work disability days over the 5‐year follow‐up period. Group‐based trajectory modelling is increasingly being applied in clinical research to map the developmental course of disease and it enabled us to identify the number, shape and size (i.e. the percentage of the population following that trajectory) of different (latent) trajectory groups in the data 18. We used Bayesian Information Criteria to evaluate model fit. In the group‐based trajectory modelling, the Bayesian Information Criterion is always negative and the maximum (the least negative value) indicates the best model 19.

Employees with diabetes in the two cohorts had the same number of distinct developmental trajectories, which were similar in shape and levels of disability; the cohorts were therefore pooled for further analysis. Associations between baseline lifestyle risk factors and the trajectory groups were examined using multivariable multinomial regression analysis with odds ratios (ORs) and their 95% CIs. The models were adjusted for age, sex, occupational grade, marital status, timing of diabetes diagnosis, comorbid disease and cohort. To determine whether the associations were different among employees with and without diabetes, we tested whether there was an interaction between diabetes status and lifestyle‐related risk factors by entering the interaction term ‘diabetes status (yes vs. no)*exposure (e.g. obesity)’ to the regression model. Similarly, we tested the interaction by sex. A three‐way interaction ‘cohort*lifestyle factor*diabetes status’ was tested to examine whether there were any differences between cohorts in these associations. A sub‐group analysis among FPSS participants was carried out to examine the cause‐specific distribution of work disability. All analyses were performed with sas 9.4 program package (sas Institute, Cary, NC, USA).

## Results

There was no difference in mean age between the FPSS and GAZEL study participants (Table S1). Diabetes was newly diagnosed in 29.1% of the FPSS and 54.0% of the GAZEL participants. The FPSS participants were less likely to have comorbid chronic diseases but more likely to be obese, while the GAZEL participants were more likely to report low physical activity, smoking, and high alcohol consumption.

During the 5‐year follow‐up, FPSS participants with and without diabetes had a median of 34.0 work disability days/5 years/person (6.8 days/year/person) and 14.0 days 5 years/person (2.8/year/person), respectively (data not shown). The largest number of disability days among people with diabetes was attributable to musculoskeletal diseases (39.1%), followed by mental and behavioural disorders (17.4%), diseases of the circulatory system (11.2%) and endocrine, nutritional and metabolic diseases (such as diabetes; 9.3%); showing that the magnitude of work‐related disability attributable to diabetes diagnosis is small. During the 5‐year follow‐up, the GAZEL participants with and without diabetes had a median of 23.0 work disability days/5 years/person (4.6 days/year/person) and 12.0 days/5 years/person (2.4 days/year/person), respectively.

**Figure 1:**
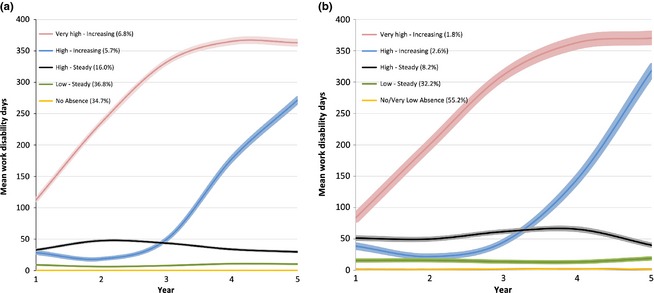
Work disability trajectories (mean days) during the 5‐year follow‐up time among (a) 1102 participants with diabetes from the Finnish Public Sector Study and (b) 500 participants with diabetes from the GAZEL study.

In the trajectory analysis, a five‐group model that had the best fit in employees with diabetes (Fig. 1) also applied to those without diabetes (Fig. S2). Three of these trajectories were associated with high disability, apart from in GAZEL participants without diabetes, where there were only two high‐disability trajectories. Average rates of disability in the high‐disability trajectories in employees with diabetes compared with controls were higher in GAZEL than in FPSS participants (Table S2), although the percentage of participants in the high‐disability categories was higher in the FPSS cohort. The two highest disability groups were collapsed in subsequent analyses because of small numbers and the combined category was labelled ‘high–increasing’. Among the GAZEL participants without diabetes, the group with ‘low–small increase’ was collapsed with the ‘low–steady’ group’. Thus, for the pooled data we used four trajectories: ‘no/very low disability’ (41.1% among diabetes cases and 48.0% among controls); ‘low–steady’ (35.4 and 34.7%, respectively; ‘high–steady’ (13.6 and 12.1%, respectively); and ‘high–increasing’ (10.0 and 5.2%, respectively; Fig 1 and Fig S2).

Table S3 shows that employees with and without diabetes in both high‐disability trajectories were more likely to be from the FPSS cohort, to be older, to be women, to have a low occupational grade, to be non‐married, to have diabetes diagnosed before baseline and to have more comorbidities and poorer health behaviours (except alcohol consumption) among all participants and smoking among non‐diabetes cases when compared with those in low‐disability trajectories.

In the multivariable adjusted models (Table 1), diabetes was associated with the ‘high–increasing’ trajectory only. Obesity was associated with ‘high–steady’ and ‘high–increasing’ trajectories among employees with and without diabetes but not the ‘low–steady’ trajectory. Low physical activity predicted ‘high–steady’ and ‘high–increasing’ trajectories among employees with diabetes and ‘high–increasing’ trajectory among employees without diabetes. There was one significant interaction between diabetes status and lifestyle risk factor; smoking was associated with ‘high–increasing’ trajectory among employees with diabetes but not among those without diabetes (*P* value for interaction = 0.015). No difference was found between disability trajectories in relation to alcohol. Further adjustment for physical activity in the model with obesity as the exposure and vice versa attenuated but did not fully explain the associations. A sensitivity analysis in which non‐drinkers were excluded did not change the null finding for alcohol use (data not shown).

**Table 1:** Multinomial logistic regression of the association between diabetes and lifestyle‐related risk factors and work disability trajectories among employees with and without diabetes

	Work disability trajectory		
	Low–steady (*n *= 1647) vs. no/very low absence (*n *= 2149)	High–steady (*n *= 590) vs. no/very low absence (*n *= 2149)	High–increasing (*n *= 322) vs. no/very low absence (*n *= 2149)
	OR (95% CI)^a^	OR (95% CI)^a^	OR (95% CI)^a^
**Diabetes**			
No	1.00	1.00	1.00
Yes	1.14 (0.99–1.32)	1.20 (0.98–1.47)	1.90 (1.47–2.46)
**Lifestyle‐related risk factors**			
* Employees with diabetes*	(*n *= 558 vs. *n *= 644)	(*n *= 213 vs. *n *= 644)	(*n *= 154 vs. *n *= 644)
Obesity			
No	1.00	1.00	1.00
Yes	1.20 (0.91–1.58)	1.82 (1.28–2.60)	1.57 (1.05–2.36)
Low physical activity			
No	1.00	1.00	1.00
Yes	1.08 (0.83–1.41)	1.69 (1.19–2.40)	2.02 (1.36–3.00)
Smoking			
No	1.00	1.00	1.00
Yes	0.80 (0.59–1.09)	0.78 (0.51–1.19)	1.88 (1.21–2.93)
High alcohol consumption			
No	1.00	1.00	1.00
Yes	0.79 (0.56–1.11)	0.83 (0.50–1.37)	1.12 (0.65–1.93)
* Employees without diabetes*	(*n *= 1089 vs. *n *= 1505)	(*n *= 377 vs. *n *= 1505)	(*n *= 168 vs. *n *= 1505)
Obesity			
No	1.00	1.00	1.00
Yes	1.32 (0.99–1.74)	1.68 (1.18–1.39)	1.88 (1.19–2.96)
Low physical activity			
No	1.00	1.00	1.00
Yes	0.86 (0.70–1.05)	1.02 (0.77–1.36)	1.78 (1.24–2.57)
Smoking			
No	1.00	1.00	1.00
Yes	1.16 (0.95–1.42)	1.19 (0.89–1.59)	1.32 (0.87–2.00)
High alcohol consumption			
No	1.00	1.00	1.00
Yes	1.11 (0.87–1.41)	0.85 (0.57–1.26)	1.14 (0.68–1.92)

OR, odds ratio.

a Adjusted for age, sex, occupational grade, marital status, timing of diabetes diagnosis (among employees with diabetes), comorbid disease and cohort.

*P* value for interaction predicting work disability trajectory: obesity and diabetes status 0.81; physical activity and diabetes status 0.19; smoking and diabetes status 0.015; alcohol use and diabetes status 0.32.

No two‐way interaction was found between men and women with regard to the association between diabetes status and disability trajectories. All three‐way interaction tests with cohort produced non‐significant findings except for alcohol consumption (*P* value = 0.001) and sex (*P* value = 0.005). Sub‐group analyses of these (Table S4) show women to have a higher risk of adverse work disability trajectory than men, with the exception of GAZEL, where no difference was found among people with diabetes. Alcohol consumption was only associated with an adverse work disability trajectory among employees with diabetes in the GAZEL cohort; however, because of small numbers, the associations were non‐significant with wide CIs.

We added up the number of lifestyle‐related risk factors that had an effect on the association, i.e. obesity, low physical activity and smoking, and found that compared with control subjects, employees with diabetes were more likely to have ≥ 2 lifestyle‐related risk factors (21.1 vs. 11.4%) and less likely to have none (40.0 vs. 57.5%; *P* value for difference < 0.001). Multivariate‐adjusted analyses (Table 2) show a strong association between an increasing number of risk factors and a more adverse disability trajectory; however, this was similar among employees with and without diabetes (*P* value for interaction = 0.56).

**Table 2:** Multinomial logistic regression of the association between the number of lifestyle‐related risk factors (obesity, low physical activity and smoking) and work disability trajectories among employees with and without diabetes

Number of lifestyle‐related risk factors		Work disability trajectory					
		Low–steady vs. no/very low absence		High–steady vs. no/very low absence		High–increasing vs. no/very low absence
	*n* in the ref. group	*n*	OR (95% CI)^a^	*n*	OR (95% CI)^a^	*n*	OR (95% CI)^a^
*Employees with diabetes*			(*n *= 508 vs. *n *= 611)		(*n *= 196 vs. *n *= 611)		(*n *= 136 vs. *n *= 611)
Risk factors							
0	276	216	1.00	57	1.00	31	1.00
1	231	197	1.03 (0.79–1.35)	83	1.50 (1.01–2.23)	54	1.74 (1.06–2.86)
2‐3	104	95	1.05 (0.74–1.48)	56	2.03 (1.28–3.22)	51	3.26 (1.90–5.57)
*Employees without diabetes*			(*n *= 989 vs. *n *= 1419)		(*n *= 353 vs. *n *= 1419)		(*n *= 146 vs. *n *= 1419)
Risk factors							
0	852	573	1.00	183	1.00	64	1.00
1	430	297	0.94 (0.78–1.13)	125	1.25 (0.95–1.63)	52	1.41 (0.95–2.11)
2‐3	137	119	1.21 (0.92–1.60)	45	1.40 (0.94–2.06)	30	2.40 (1.46–3.97)

a Adjusted for age, sex, occupational grade, marital status, timing of diabetes diagnosis (among employees with diabetes), comorbid disease and cohort.

## Discussion

In this 5‐year follow‐up study of two occupational cohorts, we analysed work disability trajectories and compared them between employees with diabetes and those without diabetes. The vast majority of employees with and without diabetes were in low‐disability trajectories. A small minority (10%) of employees with diabetes were in the most adverse ‘high–increasing’ trajectory. This percentage was double that for those without diabetes (5.2%). The multivariable adjusted model showed an association between diabetes and the ‘high–increasing’ trajectory but not the other two trajectories. Earlier studies have reported higher sickness absence levels among employees with diabetes 5, 6, 7, but the findings of the present study suggest that the majority of people with diabetes have relatively low work disability rates; a finding supported by evidence that chronic diseases, such as diabetes, have become less disabling between 1990 and 2008 20. It is also noteworthy that a considerable percentage of employees without diabetes (12% in FPSS, 28% in GAZEL) had a chronic disease other than diabetes.

The present study is probably the first to examine work disability trajectories among employees with and without diabetes. In this study, obesity was associated with both of the two high‐disability trajectories among both groups. Previous research focusing on total working populations has found increased rates of sickness absence and work disability pensions among people with unhealthy lifestyles 11, 12, 16, 21, a small, diet‐focused intervention targeting individuals with diabetes and obesity reduced disability days 22, and weight loss among people with Type 2 diabetes has improved clinical outcomes, such as glycaemic control 23. Our finding that obesity was associated with high‐disability trajectories, irrespective of diabetes status, supports earlier reports of obesity as a major cause of disease burden 24. Although exercise may especially help in maintaining glucose control in diabetes 25, we found a similar association between low physical activity and work disability trajectories among employees with and without diabetes. Obesity and low physical activity are therefore likely to be effective targets of interventions aimed at minimizing work disability among all employees.

Smoking was associated with ‘high–increasing’ trajectory among employees with diabetes but not among those without diabetes. In previous studies, smoking among patients with diabetes has been related to high blood glucose levels and insulin resistance and an acceleration of diabetes‐related complications, cardiovascular events and mortality 26. The strong association might also relate to smoking duration and intensity which were not measured in the present study. Alcohol consumption was not associated with disability trajectories, although a link between risky alcohol consumption and sickness absence has been found in other employed populations 16 as well as an association between alcohol use and poor self‐care and poor glycaemic control in diabetes 27.

As might be expected, the higher the number of lifestyle‐related risk factors, the more adverse the disability trajectory. Employees with diabetes were more likely to have ≥ 2 lifestyle‐related risk factors (21.1 vs. 11.4%) and less likely to have none (40.0 vs. 57.5%), although the association between multiple risk factors and work disability was found irrespective of diabetes status. We also found that women with and without diabetes generally had higher work disability levels than men.

A major strength of the present study is its prospective design with 5 years of follow‐up and individual, daily‐based register data on work disability measured as sickness absence and disability pension. The present study is among the first to have used group‐based trajectory membership analysis in a study of work disability. A limitation is that 5‐year consecutive data were required for each participant in order to perform trajectory analysis. In the GAZEL cohort, those who left the organization were lost to follow‐up. Another limitation of the GAZEL data is that diabetes was measured by self‐report; however, the validity of self‐reports of diabetes has been shown to be good 28. Although we adjusted our models for several confounding factors, we were not able to control for the effect of severity of disease in diabetes, treatment received or adherence to treatment, all of which may be associated with lifestyle and work disability. As in all observational studies, we cannot exclude the possibility of other unknown or unmeasured confounders or reverse causation. The number of participants in the highest disability trajectory and with multiple risk factors was relatively small. Although our models were adjusted for cohort and the cohort interaction was tested, the results cannot be assumed to be generalizable beyond them.

In conclusion, the present data suggest that the majority of employees with diabetes have low disability rates, although 10% of them are on a trajectory leading to very high disability rates. Obesity and physical inactivity predict adverse disability trajectories, irrespective of diabetes status, while smoking seems to be more important in diabetes. Clustering of lifestyle‐related risk factors is more likely in individuals with diabetes and in those with high‐disability trajectories.

## Funding sources

M.V. is supported by the Academy of Finland (258598, 265174). M.K. is supported by the Finnish Work Environment Foundation and has a professorial fellowship from the Economic and Social Research Council. J.H. is supported by the Economic and Social Research Council (ES/K01336X/1) and by the Economic and Social Research Council and Medical Research Council under the Lifelong Health and Wellbeing Cross‐Council Programme initiative [ES/L002892/1]. J.V. is supported by Era‐Age2 grant (Academy of Finland 264944). FPSS is also supported by the participating organizations. The sponsors had no role in design and conduct of the study, collection, management, analysis and interpretation of the data, or preparation, review or approval of this manuscript.

## Competing interests

None declared.

## Supporting information


**Figure S1.** Illustration of the study designs.

**Figure S2.** Work disability trajectories (mean days) during the 5‐year follow‐up time among (a) 2204 participants without diabetes from the Finnish Public Sector Study and (b) 1000 participants without diabetes from the GAZEL study.

**Table S1.** Descriptive characteristics of participants with and without diabetes at baseline in the Finnish Public Sector and the GAZEL study cohorts.

**Table S2.** Proportion of employees and mean work disability days during the 5‐year follow‐up time among employees with and without diabetes in each work disability trajectory.

**Table S3.** Descriptive characteristics of employees with and without diabetes by work disability trajectory.

**Table S4.** Multinomial logistic regression of the association of sex and alcohol use with work disability trajectory by diabetes status and cohort.

## References

[1] Standards of medical care in diabetes–2013. Diabetes Care 2013; 36 (Suppl. 1): S11–66. 23264422 10.2337/dc13-S011 PMC3537269

[2] Knol M , Twisk J , Beekman A , Heine R , Snoek F , Pouwer F . Depression as a risk factor for the onset of type 2 diabetes: a meta‐analysis. Diabetologia 2006; 49: 837–845. 16520921 10.1007/s00125-006-0159-x

[3] Fritschi C , Quinn L . Fatigue in patients with diabetes: a review. J Psychosom Res 2010; 69: 33–41. 20630261 10.1016/j.jpsychores.2010.01.021 PMC2905388

[4] Dray‐Spira R , Herquelot E , Bonenfant S , Gueguen A , Melchior M . Impact of diabetes mellitus onset on sickness absence from work–a 15‐year follow‐up of the GAZEL Occupational Cohort Study. Diabet Med 2013; 30: 549–556. 23167285 10.1111/dme.12076

[5] Kivimaki M , Vahtera J , Pentti J , Virtanen M , Elovainio M , Hemingway H . Increased sickness absence in diabetic employees: what is the role of co‐morbid conditions? Diabet Med 2007; 24: 1043–1048. 17559426 10.1111/j.1464-5491.2007.02216.x

[6] Valdmanis V , Smith DW , Page MR . Productivity and economic burden associated with diabetes. Am J Public Health 2001; 91: 129–130. 11189805 10.2105/ajph.91.1.129 PMC1446501

[7] Breton MC , Guenette L , Amiche MA , Kayibanda JF , Gregoire JP , Moisan J . Burden of diabetes on the ability to work: a systematic review. Diabetes Care 2013; 36: 740–749. 23431092 10.2337/dc12-0354 PMC3579337

[8] Herquelot E , Gueguen A , Bonenfant S , Dray‐Spira R . Impact of diabetes on work cessation: data from the GAZEL cohort. Diabetes Care 2011; 34: 1344–1349. 21562323 10.2337/dc10-2225 PMC3114330

[9] Tunceli K , Bradley CJ , Nerenz D . Keoki Williams L, Pladevall M, Elston Lafata J. The impact of diabetes on employment and work productivity. Diabetes Care 2005; 28: 2662–2667. 16249536 10.2337/diacare.28.11.2662

[10] Whiting DR , Guariguata L , Weil C , Shaw J . IDF diabetes atlas: global estimates of the prevalence of diabetes for 2011 and 2030. Diabet Res Clin Pract 2011; 94: 311–321. 10.1016/j.diabres.2011.10.029 22079683

[11] Samuelsson A , Ropponen A , Alexanderson K , Svedberg P . A prospective cohort study of disability pension due to mental diagnoses: the importance of health factors and behaviors. BMC Public Health 2013; 13: 621. 23816331 10.1186/1471-2458-13-621 PMC3733696

[12] Laaksonen M , Piha K , Martikainen P , Rahkonen O , Lahelma E . Health‐related behaviours and sickness absence from work. Occup Environ Med 2009; 66: 840–847. 19934118 10.1136/oem.2008.039248

[13] Vahtera J , Laine S , Virtanen M , Oksanen T , Koskinen A , Pentti J *et al* Employee control over working times and risk of cause‐specific disability pension: the Finnish Public Sector Study. Occup Environ Med 2010; 67: 479–485. 19914911 10.1136/oem.2008.045096 PMC3226939

[14] Marmot MG , North F , Feeney A , Head J . Alcohol consumption and sickness absence: from the Whitehall II study. Addiction 1993; 88: 369–382. 8461854 10.1111/j.1360-0443.1993.tb00824.x

[15] Ferrie JE , Head J , Shipley MJ , Vahtera J , Marmot MG , Kivimaki M . BMI, obesity, and sickness absence in the Whitehall II study. Obesity (Silver Spring) 2007; 15: 1554–1564. 17557993 10.1038/oby.2007.184

[16] Vahtera J , Poikolainen K , Kivimäki M , Ala‐Mursula L , Pentti J . Alcohol intake and sickness absence: a curvilinear relation. Am J Epidemiol 2002; 156: 969–976. 12419770 10.1093/aje/kwf138

[17] Heikkila K , Nyberg ST , Fransson EI , Alfredsson L , De Bacquer D , Bjorner JB *et al* Job strain and alcohol intake: a collaborative meta‐analysis of individual‐participant data from 140,000 men and women. PloS One 2012; 7: e40101. 22792218 10.1371/journal.pone.0040101 PMC3391232

[18] Jones BL , Nagin DS , Roeder K . A SAS procedure on mixture models for estimating developmental trajectories. Sociol Meth Res 2001; 29: 374–393.

[19] Nagin DS . Analyzing developmental trajectories: a semiparametric group‐based approach. Psychol Meth 1999; 4: 139–157.

[20] Hoeymans N , Wong A , van Gool CH , Deeg DJ , Nusselder WJ , de Klerk MM *et al* The disabling effect of diseases: a study on trends in diseases, activity limitations, and their interrelationships. Am J Public Health 2012; 102: 163–170. 22095363 10.2105/AJPH.2011.300296 PMC3490573

[21] Lahti J , Lahelma E , Rahkonen O . Changes in leisure‐time physical activity and subsequent sickness absence: a prospective cohort study among middle‐aged employees. Prev Med 2012; 55: 618–622. 23064133 10.1016/j.ypmed.2012.10.006

[22] Wolf AM , Siadaty MS , Crowther JQ , Nadler JL , Wagner DL , Cavalieri SL *et al* Impact of lifestyle intervention on lost productivity and disability: improving control with activity and nutrition. J Occup Environ Med 2009; 51: 139–145. 19209034 10.1097/JOM.0b013e3181965db5 PMC2688905

[23] Ash S , Reeves MM , Yeo S , Morrison G , Carey D , Capra S . Effect of intensive dietetic interventions on weight and glycaemic control in overweight men with Type II diabetes: a randomised trial. Int J Obes Relat Metab Disord 2003; 27: 797–802. 12821964 10.1038/sj.ijo.0802295

[24] Muller‐Riemenschneider F , Reinhold T , Berghofer A , Willich SN . Health‐economic burden of obesity in Europe. Eur J Epidemiol 2008; 23: 499–509. 18509729 10.1007/s10654-008-9239-1

[25] Umpierre D , Ribeiro PA , Kramer CK , Leitao CB , Zucatti AT , Azevedo MJ *et al* Physical activity advice only or structured exercise training and association with HbA1c levels in type 2 diabetes: a systematic review and meta‐analysis. JAMA 2011; 305: 1790–1799. 21540423 10.1001/jama.2011.576

[26] Tonstad S . Cigarette smoking, smoking cessation, and diabetes. Diabet Res Clin Pract 2009; 85: 4–13. 10.1016/j.diabres.2009.04.013 19427049

[27] Engler PA , Ramsey SE , Smith RJ . Alcohol use of diabetes patients: the need for assessment and intervention. Acta Diabetol 2013; 50: 93–99. 20532803 10.1007/s00592-010-0200-x PMC2954251

[28] Oksanen T , Kivimaki M , Pentti J , Virtanen M , Klaukka T , Vahtera J . Self‐report as an indicator of incident disease. Ann Epidemiol 2010; 20: 547–554. 20538198 10.1016/j.annepidem.2010.03.017

