# Supplementary material for: Lifestyle‐related risk factors and trajectories of work disability over 5 years in employees with diabetes: findings from two prospective cohort studies
Source: Diabet Med. 2015 May 15;32(10):1335–41. doi: 10.1111/dme.12787 (PMC4975699; doi:10.1111/dme.12787)
Supplement: Supplementary file 1 — Figure S1. Illustration of the study designs. [file DME-32-1335-s001.docx]

**Supplemental Table 1** Descriptive characteristics of participants with and without diabetes at baseline in the Finnish Public Sector and the GAZEL study cohorts

|  | Employees with diabetes | | |  | Employees without diabetes | | | |
| --- | --- | --- | --- | --- | --- | --- | --- | --- |
|  | Finnish Public Sector Study (n=1,102) | GAZEL Study (n=500) | *P** | | | Finnish Public Sector Study (n=2,204) | GAZEL Study (n=1,000) | *P** |
| Age (mean, SD) | 46.4 (8.6) | 47.0 (3.3) | 0.10 | | | 46.3 (8.5) | 46.6 (3.1) | 0.31 |
| Sex, *n* (%) |  |  | <.0001 | | |  |  | <.0001 |
| Men | 293 (26.6) | 385 (77.0) |  | | | 586 (26.6) | 770 (77.0) |  |
| Women | 809 (73.4) | 115 (23.0) |  | | | 1,618 (73.4) | 230 (23.0) |  |
| Occupational grade, *n* (%) |  |  | <.0001 | | |  |  | <.0001 |
| High | 270 (24.5) | 171 (34.3) |  | | | 540 (24.5) | 342 (34.2) |  |
| Intermediate | 586 (53.2) | 250 (50.1) |  | | | 1,174 (53.3) | 502 (50.2) |  |
| Low | 245 (22.3) | 78 (15.6) |  | | | 490 (22.2) | 156 (15.6) |  |
| Marital status, *n* (%) |  |  | <.0001 | | |  |  | <.0001 |
| Married/cohabiting | 809 (74.4) | 431 (86.4) |  | | | 1,648 (74.8) | 864 (86.4) |  |
| Non-married/cohabiting | 278 (25.6) | 68 (13.6) |  | | | 556 (25.2) | 136 (13.6) |  |
| Timing of diabetes diagnosis, *n* (%) |  |  | <.0001 | | |  |  | n.a. |
| Previous† | 781 (70.9) | 230 (46.0) |  | | | n.a. | n.a. |  |
| New† | 321 (29.1) | 270 (54.0) |  | | | n.a. | n.a. |  |
| Comorbid disease, *n* (%) |  |  | 0.010 | | |  |  | <.0001 |
| No | 729 (66.2) | 288 (59.4) |  | | | 1,933 (87.7) | 671 (71.8) |  |
| Yes | 373 (33.9) | 197 (40.6) |  | | | 271 (12.3) | 264 (28.2) |  |
| Obesity, *n* (%) |  |  | <.0001 | | |  |  | <.0001 |
| No | 699 (66.1) | 360 (76.4) |  | | | 1,857 (86.3) | 874 (97.1) |  |
| Yes | 359 (33.9) | 111 (23.6) |  | | | 294 (13.7) | 26 (2.9) |  |
| Low physical activity, *n* (%) |  |  | <.0001 | | |  |  | <.0001 |
| No | 761 (69.7) | 266 (58.9) |  | | | 1,740 (79.7) | 610 (69.2) |  |
| Yes | 331 (30.3) | 186 (41.2) |  | | | 442 (20.3) | 271 (30.8) |  |
| Smoking, *n* (%) |  |  | 0.041 | | |  |  | 0.0002 |
| No | 853 (80.3) | 379 (75.8) |  | | | 1,719 (80.2) | 736 (74.3) |  |
| Yes | 209 (19.7) | 121 (24.2) |  | | | 425 (19.8) | 255 (25.7) |  |
| High alcohol use, *n* (%) |  |  | <.0001 | | |  |  | <.0001 |
| No | 948 (86.8) | 337 (78.7) |  | | | 1,953 (89.0) | 692 (80.8) |  |
| Yes | 144 (13.2) | 91 (21.3) |  | | | 241 (11.0) | 164 (19.2) |  |

**P-*values for difference between cohorts are from the Χ^2^ tests and the univariate analysis of variance.

†In the Finnish Public Sector Study, new indicates diabetes detected at study baseline and previous indicates diabetes detected from registers before study baseline. In the GAZEL, new indicates diabetes detected after the study outset (survey 1) and previous indicates diabetes detected at survey 1.
