# Supplementary material for: Lifestyle‐related risk factors and trajectories of work disability over 5 years in employees with diabetes: findings from two prospective cohort studies
Source: Diabet Med. 2015 May 15;32(10):1335–41. doi: 10.1111/dme.12787 (PMC4975699; doi:10.1111/dme.12787)
Supplement: Supplementary file 2 — Figure S2. Work disability trajectories (mean days) during the 5‐year follow‐up time among (a) 2204 participants without diabetes from the Finnish Public Sector Study and (b) 1000 participants without diabetes from the GAZEL study. [file DME-32-1335-s002.docx]

**Supplemental Table 2** Proportion of employees and mean work disability days during the 5-year follow-up time among employees with and without diabetes in each work disability trajectory

|  |  | Finnish Public Sector Study | | | |  | GAZEL Study | | | |
| --- | --- | --- | --- | --- | --- | --- | --- | --- | --- | --- |
| Trajectory | Trajectory in the final analyses | Employees with diabetes (n=1,102) | | Employees without diabetes (n=2,204) | |  | Employees with diabetes (n=500) | | Employees without diabetes (n=1,000) | |
|  |  | *n (*%) | Mean | *n* (%) | Mean |  | *n* (%) | Mean | *n* (%) | Mean |
| No/ very low absence | No/ very low absence | 382 (34.7) | 0.0 | 1,022 (46.4) | 0.0 |  | 276 (55.2) | 1.7 | 516 (51.6) | 0.6 |
| Low - steady | Low - steady | 406 (36.8) | 8.9 | 720 (32.7) | 7.8 |  | 161 (32.2) | 15.5 | 210 (21.0) | 8.2 |
| Low – small increase | Low - steady | - | - | - | - |  | - | - | 182 (18.2) | 10.8 |
| High - steady | High - steady | 176 (16.0) | 37.6 | 315 (14.3) | 32.2 |  | 41 (8.2) | 53.7 | 71 (7.1) | 42.3 |
| High - increasing | High - increasing | 63 (5.7) | 109.2 | 83 (3.8) | 113.3 |  | 13 (2.6) | 113.8 | - | - |
| Very high - increasing | High - increasing | 75 (6.8) | 281.6 | 64 (2.9) | 301.4 |  | 9 (1.8) | 264.8 | 21 (2.1) | 166.5 |
