# Supplementary material for: Lifestyle‐related risk factors and trajectories of work disability over 5 years in employees with diabetes: findings from two prospective cohort studies
Source: Diabet Med. 2015 May 15;32(10):1335–41. doi: 10.1111/dme.12787 (PMC4975699; doi:10.1111/dme.12787)
Supplement: Supplementary file 3 — Table S1. Descriptive characteristics of participants with and without diabetes at baseline in the Finnish Public Sector and the GAZEL study cohorts. [file DME-32-1335-s003.docx]

**Supplemental Table 3** Descriptive characteristics of employees with and without diabetes by work disability trajectory

| Employees with diabetes | All (*n*=1,602) | No/very low absence (*n*=658) | Low – steady (*n*=567) | High – steady (*n*=217) | High – increasing (*n*=160) | *P** |
| --- | --- | --- | --- | --- | --- | --- |
| Cohort, *n* (%) |  |  |  |  |  | <.0001 |
| FPSS | 1,102 (68.8) | 382 (58.1) | 406 (71.6) | 176 (81.1) | 138 (86.3) |  |
| GAZEL | 500 (32.2) | 276 (42.0) | 161 (28.4) | 41 (18.9) | 22 (13.8) |  |
| Age (mean, SD) | 46.6 (7.4) | 46.2 (7.1) | 45.7 (7.9) | 47.2 (7.3) | 50.3 (6.0) | <.0001 |
| Sex, *n* (%) |  |  |  |  |  | <.0001 |
| Men | 678 (42.3) | 346 (52.6) | 209 (36.9) | 66 (30.4) | 57 (35.6) |  |
| Women | 924 (57.7) | 312 (47.4) | 358 (63.1) | 151 (69.6) | 103 (64.4) |  |
| Occupational grade, *n* (%) |  |  |  |  |  | <.0001 |
| High | 441 (27.6) | 262 (39.9) | 136 (24.0) | 22 (10.1) | 21 (13.2) |  |
| Intermediate | 836 (52.3) | 324 (49.3) | 305 (53.8) | 129 (59.5) | 78 (49.1) |  |
| Low | 323 (20.2) | 71 (10.8) | 126 (22.2) | 66 (30.4) | 60 (37.7) |  |
| Marital status, *n* (%) |  |  |  |  |  | 0.023 |
| Married/cohabiting | 1,240 (78.2) | 524 (80.3) | 445 (79.0) | 150 (70.4) | 121 (77.1) |  |
| Non-married/cohabiting | 346 (21.8) | 129 (19.8) | 118 (21.0) | 63 (29.6) | 36 (22.9) |  |
| Timing of diabetes diagnosis, *n* (%) |  |  |  |  |  | 0.019 |
| Previous† | 1,011 (63.1) | 389 (59.1) | 363 (64.0) | 151 (69.6) | 108 (67.5) |  |
| New† | 591 (36.9) | 269 (40.9) | 204 (36.0) | 66 (30.4) | 52 (32.5) |  |
| Comorbid disease, *n* (%) |  |  |  |  |  | <.0001 |
| No | 1,017 (64.1) | 457 (70.3) | 355 (63.2) | 120 (55.3) | 85 (53.8) |  |
| Yes | 570 (35.9) | 193 (29.7) | 207 (36.8) | 97 (44.7) | 73 (46.2) |  |
| Obesity, *n* (%) |  |  |  |  |  | <.0001 |
| No | 1,059 (69.3) | 482 (76.2) | 370 (69.0) | 119 (56.9) | 88 (58.3) |  |
| Yes | 470 (30.7) | 151 (23.9) | 166 (31.0) | 90 (43.1) | 63 (41.7) |  |
| Low physical activity, *n* (%) |  |  |  |  |  | <.0001 |
| No | 1,027 (66.5) | 442 (69.7) | 377 (69.7) | 125 (58.7) | 83 (53.2) |  |
| Yes | 517 (33.5) | 192 (30.3) | 164 (30.3) | 88 (41.3) | 73 (46.8) |  |
| Smoking, *n* (%) |  |  |  |  |  | 0.003 |
| No | 1,232 (78.9) | 509 (78.7) | 452 (81.4) | 169 (80.9) | 102 (67.6) |  |
| Yes | 330 (21.1) | 138 (21.3) | 103 (18.6) | 40 (19.1) | 49 (32.5) |  |
| High alcohol use, *n* (%) |  |  |  |  |  | 0.057 |
| No | 1,285 (84.5) | 505 (81.6) | 474 (87.0) | 180 (87.0) | 126 (84.6) |  |
| Yes | 235 (15.5) | 114 (18.4) | 71 (13.0) | 27 (13.0) | 23 (15.4) |  |
|  |  |  |  |  |  |  |
| Employees without diabetes | All (*n*=3,204) | None/very low (*n*=1,538) | Low – steady (*n*=1,112) | High – steady (*n*=386) | High – increasing (*n*=168) | *P** |
| Cohort, *n* (%) |  |  |  |  |  | <.0001 |
| FPSS | 2,204 (68.9) | 1,022 (66.5) | 720 (64.8) | 315 (81.6) | 147 (87.5) |  |
| GAZEL | 1,000 (31.2) | 516 (33.6) | 392 (35.3) | 71 (18.4) | 21 (12.5) |  |
| Age (mean, SD) | 46.4 (7.2) | 46.2 (7.1) | 45.9 (7.1) | 47.0 (7.7) | 50.6 (6.3) | <.0001 |
| Sex, *n* (%) |  |  |  |  |  | <.0001 |
| Men | 1,356 (42.3) | 768 (49.9) | 425 (38.2) | 108 (28.0) | 55 (32.7) |  |
| Women | 1,848 (57.7) | 770 (50.1) | 687 (61.8) | 278 (72.0) | 113 (67.3) |  |
| Occupational grade, *n* (%) |  |  |  |  |  | <.0001 |
| High | 882 (27.5) | 579 (37.7) | 240 (21.6) | 49 (12.7) | 14 (8.3) |  |
| Intermediate | 1,676 (52.3) | 737 (47.9) | 624 (56.1) | 225 (58.3) | 90 (53.6) |  |
| Low | 646 (20.2) | 222 (14.4) | 248 (22.3) | 112 (29.0) | 64 (38.1) |  |
| Marital status, *n* (%) |  |  |  |  |  | 0.06 |
| Married/cohabiting | 2,512 (78.4) | 1,234 (80.2) | 855 (76.9) | 289 (74.9) | 134 (79.8) |  |
| Non-married/cohabiting | 692 (21.6) | 304 (19.8) | 257 (23.1) | 97 (25.1) | 34 (20.2) |  |
| Comorbid disease, *n* (%) |  |  |  |  |  | <.0001 |
| No | 2,604 (83.0) | 1321 (87.8) | 881 (80.9) | 288 (76.4) | 114 (67.9) |  |
| Yes | 535 (17.0) | 184 (12.2) | 208 (19.1) | 89 (23.6) | 54 (32.1) |  |
| Obesity, *n* (%) |  |  |  |  |  | <.0001 |
| No | 2731 (89.5) | 1,357 (92.0) | 940 (89.4) | 307 (83.9) | 127 (79.9) |  |
| Yes | 320 (10.5) | 118 (8.0) | 111 (10.6) | 59 (16.1) | 32 (20.1) |  |
| Low physical activity, *n* (%) |  |  |  |  |  | 0.0003 |
| No | 2,350 (76.7) | 1,143 (77.7) | 822 (77.7) | 283 (76.1) | 102 (63.0) |  |
| Yes | 713 (23.3) | 328 (22.3) | 236 (22.3) | 89 (23.9) | 60 (37.0) |  |
| Smoking, *n* (%) |  |  |  |  |  | 0.48 |
| No | 2,455 (78.3) | 1,206 (79.5) | 834 (77.2) | 293 (77.3) | 122 (76.7) |  |
| Yes | 680 (21.7) | 311 (20.5) | 246 (22.8) | 86 (22.7) | 37 (23.3) |  |
| High alcohol use, *n* (%) |  |  |  |  |  | 0.11 |
| No | 2,645 (86.7) | 1,257 (85.7) | 908 (86.6) | 336 (90.6) | 144 (87.3) |  |
| Yes | 405 (13.3) | 209 (14.3) | 140 (13.4) | 35 (9.4) | 21 (12.7) |  |

**P-*values for difference between cohorts are from the Χ^2^ tests and the univariate analysis of variance.

†In the Finnish Public Sector Study, new indicates diabetes detected at study baseline and previous indicates diabetes detected from registers before study baseline. In the GAZEL, new indicates diabetes detected after the study outset (survey 1) and previous indicates diabetes detected at survey 1.
