# Supplementary material for: Lifestyle‐related risk factors and trajectories of work disability over 5 years in employees with diabetes: findings from two prospective cohort studies
Source: Diabet Med. 2015 May 15;32(10):1335–41. doi: 10.1111/dme.12787 (PMC4975699; doi:10.1111/dme.12787)
Supplement: Supplementary file 4 — Table S2. Proportion of employees and mean work disability days during the 5‐year follow‐up time among employees with and without diabetes in each work disability trajectory. [file DME-32-1335-s004.docx]

**Supplemental Table 4** Multinomial logistic regression of the association of sex and alcohol use with work disability trajectory by diabetes status and cohort.

|  | Work disability trajectory | | | | |
| --- | --- | --- | --- | --- | --- |
|  | Low – steady vs no/very low absence |  | High – steady vs no/very low absence |  | High – increasing vs no/very low absence |
|  | OR (95% CI)* |  | OR (95% CI)* |  | OR (95% CI)* |
| Sex and diabetes status |  |  |  |  |  |
| Finnish Public Sector Study |  |  |  |  |  |
| *Employees with diabetes* (*n*=1,086) | (*n*=402 *vs* *n*=378) |  | (*n*=172 *vs* *n*=378) |  | (*n*=134 *vs* *n*=378) |
| Men (*n*=286) | 1.00 |  | 1.00 |  | 1.00 |
| Women (*n*=800) | 1.23 (0.87-1.73) |  | 1.92 (1.19-3.09) |  | 1.60 (0.98-2.59) |
| *Employees without diabetes* (*n*=2,204) | (*n*=720 *vs* *n*=1,022) |  | (*n*=315 *vs* *n*=1,022) |  | (*n*=147 *vs* *n*=1,022) |
| Men (*n*=586) | 1.00 |  | 1.00 |  | 1.00 |
| Women (*n*=1,618) | 1.86 (1.46-2.36) |  | 1.93 (1.39-2.66) |  | 1.62 (1.07-2.46) |
| GAZEL Study |  |  |  |  |  |
| *Employees with diabetes* (*n*=483) | (*n*=156 *vs* *n*=266) |  | (*n*=41 *vs* *n*=266) |  | (*n*=20 *vs* *n*=266) |
| Men (*n*=372) | 1.00 |  | 1.00 |  | 1.00 |
| Women (*n*=111) | 2.87 (1.70-4.84) |  | 1.04 (0.43-2.50) |  | 1.16 (0.35-3.80) |
| *Employees without diabetes* (*n*=935) | (*n*=369 vs *n*=483) |  | (*n*=62 vs *n*=483) |  | (*n*=21 vs *n*=483) |
| Men (*n*=724) | 1.00 |  | 1.00 |  | 1.00 |
| Women (*n*=211) | 2.01 (1.39-2.90) |  | 3.60 (1.96-6.62) |  | 3.06 (1.10-8.52) |
| Alcohol use and diabetes status |  |  |  |  |  |
| Finnish Public Sector Study |  |  |  |  |  |
| *Employees with diabetes* (*n*=1,080) |  |  |  |  |  |
| High alcohol use | (*n*=401 *vs* *n*=378) |  | (*n*=172 *vs* *n*=378) |  | (*n*=129 *vs* *n*=378) |
| No (*n*=939) | 1.00 |  | 1.00 |  | 1.00 |
| Yes (*n*=141) | 0.85 (0.55-1.31) |  | 0.63 (0.33-1.18) |  | 0.97 (0.52-1.79) |
| *Employees without diabetes* (*n*=2,194) |  |  |  |  |  |
| High alcohol use | (*n*=716 *vs* *n*=1,018) |  | (*n*=313 *vs* *n*=1,018) |  | (*n*=147 *vs* *n*=1,018) |
| No (*n*=1,953) | 1.00 |  | 1.00 |  | 1.00 |
| Yes (*n*=241) | 0.94 (0.68-1.30) |  | 0.95 (0.61-1.48) |  | 1.27 (0.74-2.20) |
| GAZEL Study |  |  |  |  |  |
| *Employees with diabetes* (*n*=420) |  |  |  |  |  |
| High alcohol use | (*n*=139 *vs* *n*=234) |  | (*n*=32 *vs* *n*=234) |  | (*n*=15 *vs* *n*=234) |
| No (*n*=330) | 1.00 |  | 1.00 |  | 1.00 |
| Yes (*n*=90) | 0.66 (0.37-1.19) |  | 1.76 (0.74-4.19) |  | 2.22 (0.65-7.57) |
| *Employees without diabetes* (*n*=822) |  |  |  |  |  |
| High alcohol use | (*n*=321 *vs* *n*=430) |  | (*n*=53 *vs* *n*=430) |  | (*n*=18 *vs* *n*=430) |
| No (*n*=666) | 1.00 |  | 1.00 |  | 1.00 |
| Yes (*n*=156) | 1.34 (0.92-1.95) |  | 0.42 (0.14-1.22) |  | 0.36 (0.05-2.89) |

*Adjusted for age, sex (when relevant), occupational grade, marital status, timing of diabetes diagnosis (among employees with diabetes), and comorbid disease.
