# Supplementary figures and images for: Lifestyle‐related risk factors and trajectories of work disability over 5 years in employees with diabetes: findings from two prospective cohort studies
Source: Diabet Med. 2015 May 15;32(10):1335–41. doi: 10.1111/dme.12787 (PMC4975699; doi:10.1111/dme.12787)

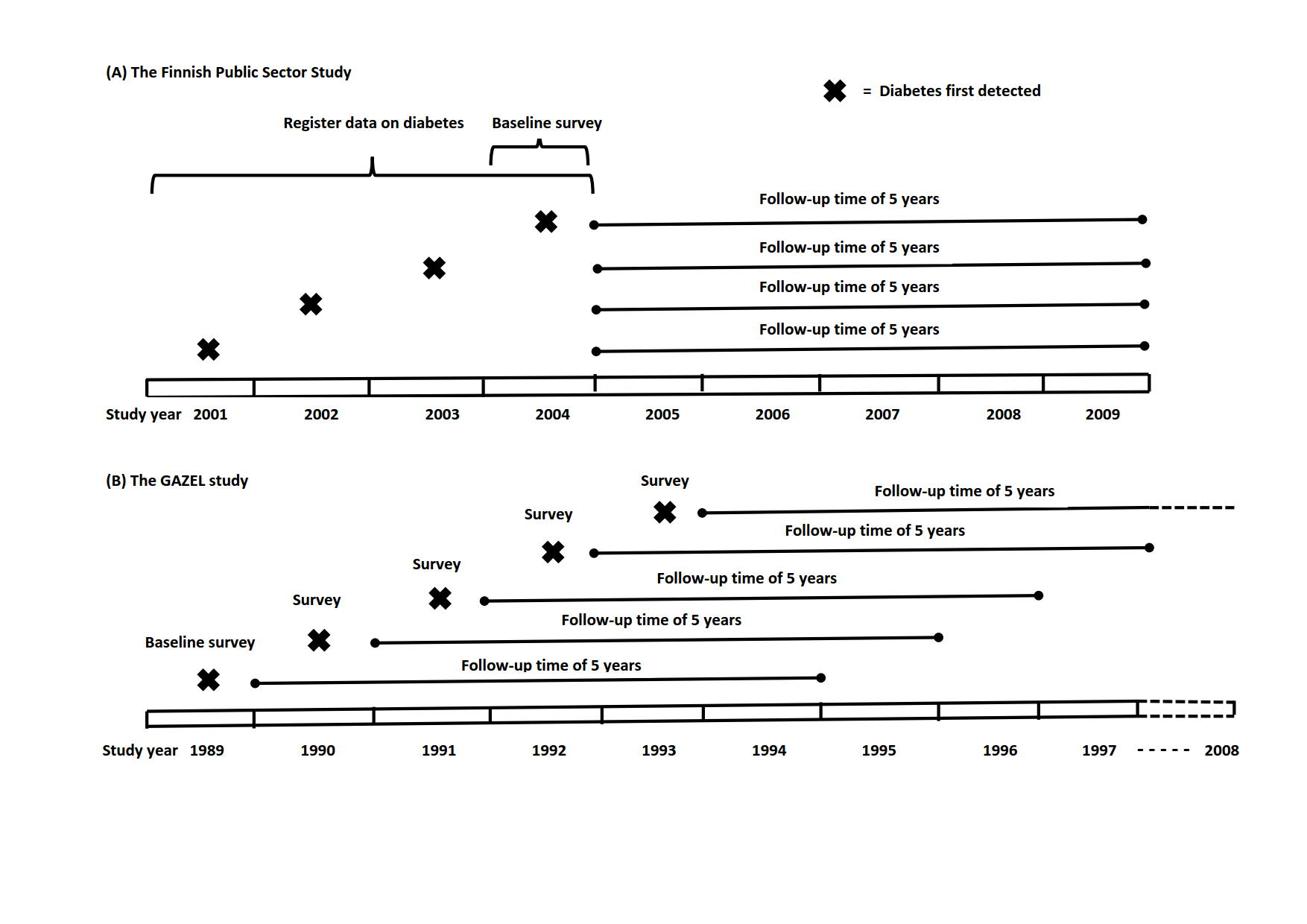

Supplement: Supplementary file 5 — Table S3. Descriptive characteristics of employees with and without diabetes by work disability trajectory. [file DME-32-1335-s005.jpg]

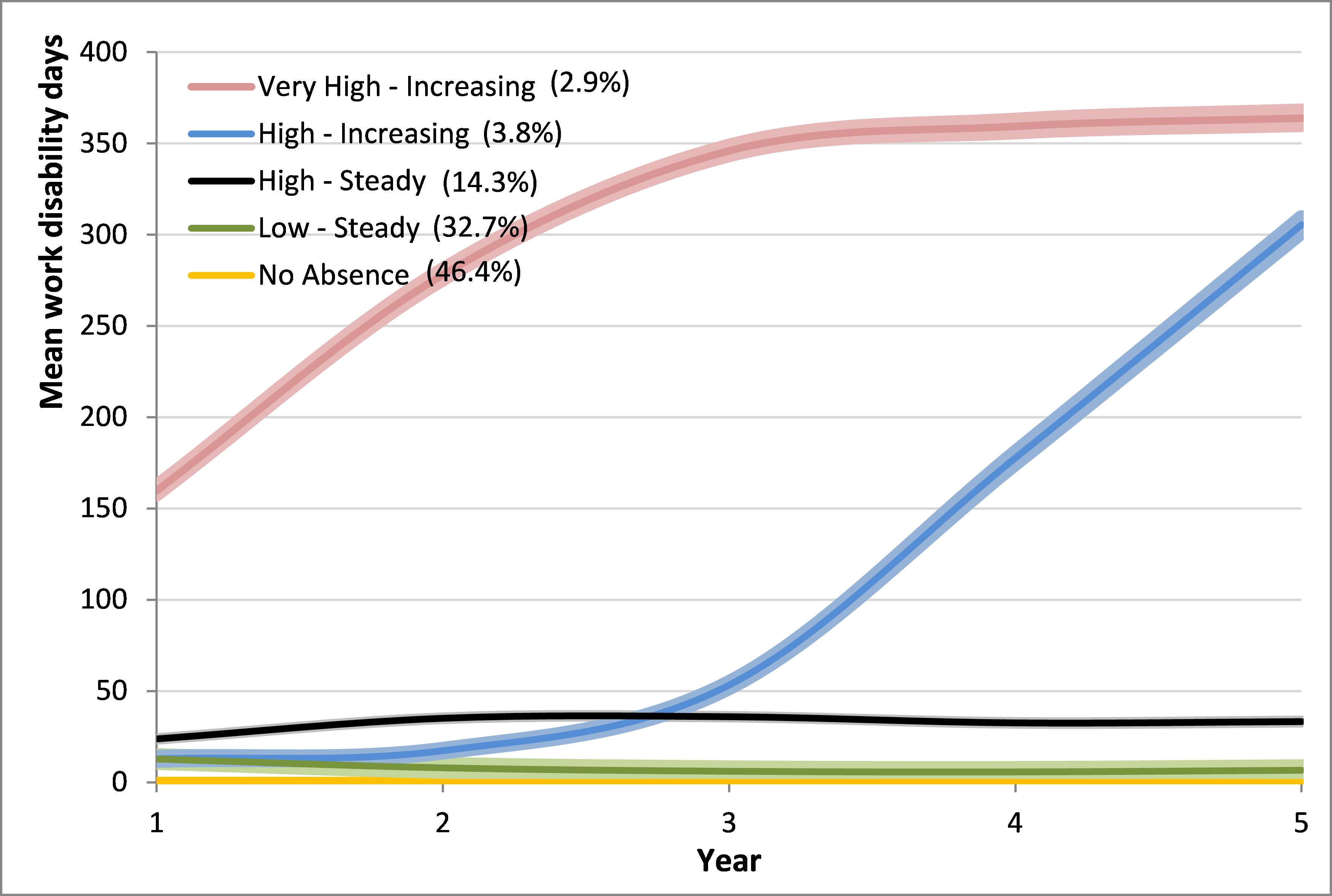

Supplement: Supplementary file 6 — Table S4. Multinomial logistic regression of the association of sex and alcohol use with work disability trajectory by diabetes status and cohort. [file DME-32-1335-s006.jpg]

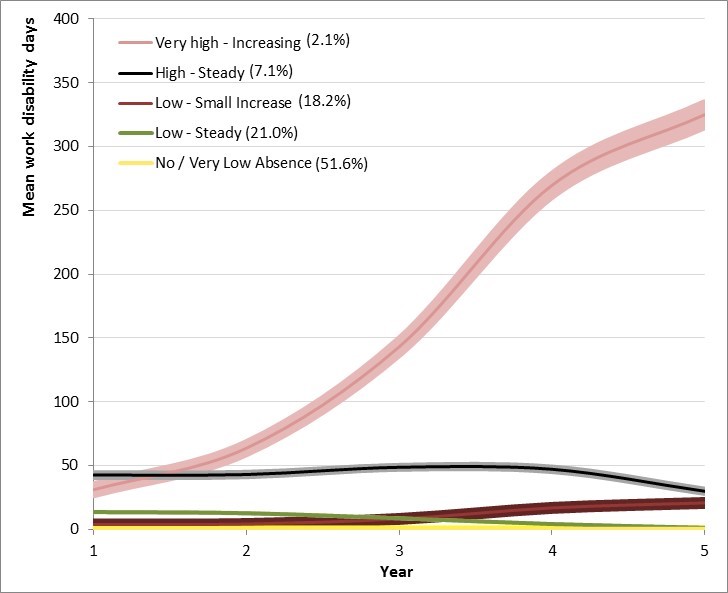

Supplement: Supplementary file 7 [file DME-32-1335-s007.jpg]
